# Molecular Dynamics Simulations Reveal How Competing Protein–Surface Interactions for Glycine, Citrate, and Water Modulate Stability in Antibody Fragment Formulations

**DOI:** 10.1021/acs.molpharmaceut.4c00332

**Published:** 2024-10-21

**Authors:** Akash Pandya, Cheng Zhang, Teresa S. Barata, Steve Brocchini, Mark J. Howard, Mire Zloh, Paul A. Dalby

**Affiliations:** † Department of Biochemical Engineering, University College London, Gower Street, London WC1E 6BT, U.K.; ‡ School of Pharmacy, University College London, 29-39 Brunswick Square, London WC1N 1AX, U.K.; § School of Chemistry, University of Leeds, Leeds LS2 9JT, U.K.

**Keywords:** Fab, formulation, stability, aggregation, melting temperature, enthalpy change, preferential interaction

## Abstract

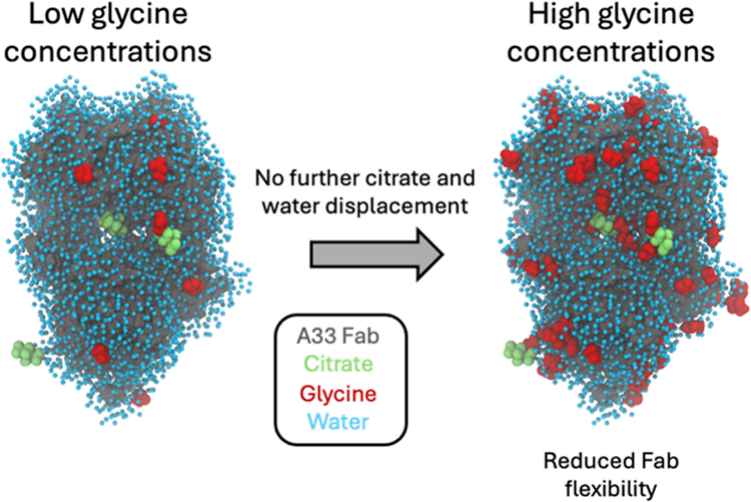

The design of stable formulations remains a major challenge for protein therapeutics, particularly the need to minimize aggregation. Experimental formulation screens are typically based on thermal transition midpoints (*T*_m_), and forced degradation studies at elevated temperatures. Both approaches give limited predictions of long-term storage stability, particularly at low temperatures. Better understanding of the mechanisms of action for formulation of excipients and buffers could lead to improved strategies for formulation design. Here, we identified a complex impact of glycine concentration on the experimentally determined stability of an antibody Fab fragment and then used molecular dynamics simulations to reveal mechanisms that underpin these complex behaviors. *T*_m_ values increased monotonically with glycine concentration, but associated Δ*S*_vh_ measurements revealed more complex changes in the native ensemble dynamics, which reached a maximum at 30 mg/mL. The aggregation kinetics at 65 °C were similar at 0 and 20 mg/mL glycine, but then significantly slower at 50 mg/mL. These complex behaviors indicated changes in the dominant stabilizing mechanisms as the glycine concentration was increased. MD revealed a complex balance of glycine self-interaction, and differentially preferred interactions of glycine with the Fab as it displaced hydration-shell water, and surface-bound water and citrate buffer molecules. As a result, glycine binding to the Fab surface had different effects at different concentrations, and led from preferential interactions at low concentrations to preferential exclusion at higher concentrations. During preferential interaction, glycine displaced water from the Fab hydration shell, and a small number of water and citrate molecules from the Fab surface, which reduced the protein dynamics as measured by root-mean-square fluctuation (RMSF) on the short time scales of MD. By contrast, the native ensemble dynamics increased according to Δ*S*_vh_, suggesting increased conformational changes on longer time scales. The aggregation kinetics did not change at low glycine concentrations, and so the opposing dynamics effects either canceled out or were not directly relevant to aggregation. During preferential exclusion at higher glycine concentrations, glycine could only bind to the Fab surface through the displacement of citrate buffer molecules already favorably bound on the Fab surface. Displacement of citrate increased the flexibility (RMSF) of the Fab, as glycine formed fewer bridging hydrogen bonds to the Fab surface. Overall, the slowing of aggregation kinetics coincided with reduced flexibility in the Fab ensemble at the very highest glycine concentrations, as determined by both RMSF and Δ*S*_vh_, and occurred at a point where glycine binding displaced neither water nor citrate. These final interactions with the Fab surface were driven by mass action and were the least favorable, leading to a macromolecular crowding effect under the regime of preferential exclusion that stabilized the dynamics of Fab.

## Introduction

Achieving liquid formulations that are stable for long periods during storage and transport, often at 4–8 °C, is critical to the successful development of therapeutic proteins such as antibodies and other antibody-based formats.^1−5^ Protein aggregation is a major degradation pathway that can lead to loss of potency and increased risk of adverse immunogenicity.^6,7^ While a formulation must be demonstrated to be stable often for a year or more, this is not a practical time scale for formulation development. Faster approaches for design and optimization include high throughput measurements of thermal transition midpoints (*T*_m_), and forced degradation at elevated temperature or agitation.^8−10^ While *T*_m_ measurements are convenient, they do not always correlate well with aggregation kinetics.^11−13^ In such cases, the global unfolding of proteins, as probed by *T*_m_ measurements, may not be a rate limiting step in the formation of aggregates. Instead, aggregation is often driven by partial unfolding events to intermediate states or even within the native ensemble, especially at low storage temperatures that do not promote global unfolding.^9,12−15^ Therefore, it cannot always be guaranteed that minimizing global unfolding (or maximizing *T*_m_), would also minimize the partial unfolding events that lead to aggregation.^9^

Accelerated degradation studies fare better when carefully designed, using a range of elevated temperatures to obtain aggregation kinetics that can be extrapolated back to lower storage temperatures.^9^ However, this approach is still often undermined by nonlinear Arrhenius behavior, indicating changes in the aggregation mechanism across the temperature range used.^9,12,16^ This can be corrected by using nonlinear Arrhenius models,^17^ but can still take several months to obtain the required aggregation kinetics. With these challenges, alongside time pressure to develop formulations rapidly, there remains a need for more efficient and predictable approaches to formulating therapeutic proteins. This, in turn, is driving the need to better understand degradation mechanisms such as aggregation as well as the mechanisms of action for typical formulation components. Such advances could lead to improved formulation approaches.

Protein aggregation typically involves at least two steps, conformational changes occurring in the native state and self-association into higher order aggregates.^14,15^ Conformational changes can either be global or structurally localized events, leading to the exposure of buried aggregation-prone regions (APRs), and subsequent formation of non-native dimers and/or oligomers.^14,18^ Several algorithms aim to predict such APRs based on amino acid sequences, including AGGRESCAN,^19^ TANGO,^20^ and PASTA.^21^ Other algorithms have extended these approaches to account for structure, such as Camsol,^22^ and structural dynamics that may expose the APRs, such as AGGRESCAN three-dimensional (3D)^23^ and the spatial aggregation propensity (SAP) tool.^24^ Such tools can inform the design of potential mutations to improve the stability of proteins.^25−27^ However, it is still challenging to use them to inform the design of formulations because the impact of specific formulations on local protein dynamics is not so well understood. In addition, the kinetics of self-association of two or more proteins to form aggregates is also dependent on protein surface hydrophobicity,^22,28^ protein concentration,^29^ and other solution conditions that modify the protein surface charge and viscosity.^30,31^

Preferential interaction/exclusion theory is widely used to explain excipient behavior in formulations.^32−34^ In simple terms, an excipient is deemed to be preferentially interacting with a protein when the concentration of the excipient close to the protein surface (e.g., in the first and second hydration layers) is higher than in the bulk solvent. For preferential exclusion, the excipient concentration close to the protein is lower than that in the bulk solvent. Preferential exclusion occurs when the protein has a higher affinity for water (or any other cosolvents present) than for the excipient. This increases the chemical potential of the excipient, as it is excluded from the protein hydration shell. If this effect is stronger for the globally, or partially unfolded states, than for the native protein, then the protein will be stabilized into a compact native form. Additionally, increased stability is related to increased surface tension, which arises from the increased chemical potential of an excipient excluded from the protein hydration shell. In this view, it is more thermodynamically unfavorable to create a cavity in the solvent into which the protein can (partially) unfold. Macromolecular crowding also plays a role whereby the partial specific volume available for a protein to unfold into is reduced.^35^ Preferential interaction, in which the protein prefers to interact with the excipient, is also thought to play a stabilizing role, provided that the excipient does not also prefer to interact with buried, often hydrophobic residues, favoring the unfolded state(s).^36^ Such interactions would be highly specific to each protein and excipient combination, so it is difficult to predict the role of preferential interactions for each formulation, let alone the relative balance of these effects with preferential exclusion.

Docking tools have been used previously to evaluate potential excipient interactions with proteins, and identified three key hotspots in A33 Fab which tended to interact with eight excipients tested.^37^ The highest calculated binding energies showed a potential correlation with thermal stability measurements (*T*_m_), although this could not directly account for differences in the excipient concentration or protein dynamics.

Molecular dynamics simulations have also been used to evaluate excipient interactions with proteins.^38−42^ Simulations with arginine revealed a tendency to form hydrogen-bonded clusters with other arginine molecules, which influenced its aggregation inhibition properties. Due to self-association, arginine was found to be preferentially excluded from the protein surface, especially at higher bulk arginine concentrations after the available binding sites on the protein surface had been saturated.^43,44^ The arginine clusters effectively crowded out protein–protein interactions, while cation-π interactions were found to stabilize the unfolded intermediates. Meanwhile, MD simulations of three mAb molecules with sorbitol, sucrose, and trehalose showed all to be preferentially excluded from the mAb surfaces, but also having considerable local interactions especially with exposed hydrophobic residues, that could potentially shield the mAbs from self-interaction.^38^

A33 Fab is a therapeutically relevant humanized antibody fragment^45^ for which we have extensively studied its aggregation kinetics, thermal unfolding, and structural changes for a wide range of pH, ionic strength, and temperatures.^12^ The *T*_m_ values were found to correlate with aggregation kinetics at 45–65 °C, but not at ambient temperatures and below, while biophysical studies using small-angle X-ray scattering, single molecule Forster resonance energy transfer (smFRET), and molecular dynamics simulations have identified partial unfolding of the C_L_ domain under aggregation-prone conditions.^46,47^ Meanwhile, Rosetta-designed single mutants in the C_L_ and C_H_ domains aimed at reducing flexibility, which led to slowed aggregation kinetics at 65 °C in cases that also increased the vant Hoff entropy of unfolding Δ*S*_vh_ at the thermal transition midpoints, with no change in *T*_m_. This indicated that a more compact native ensemble led to reduced aggregation propensity, while kinetic modeling revealed A33 Fab aggregation kinetics to be rate limited by partial unfolding to near native states, N*. Thus, the aggregation kinetics at 65 °C are dependent on a combination of global conformational stability (affecting *T*_m_) and changes in local conformational flexibility in the native ensemble (affecting Δ*S*_vh_), and that this balance is readily shifted by the specific formulations being tested. This model was also supported by kinetic models that explained the concentration-dependent behavior of A33 Fab aggregation such that the reversible formation of N* from N was suppressed at high protein concentrations.^29^

Here, we investigate the complex dependence on glycine concentration for the stability of A33 Fab in citrate buffer, as measured by *T*_m_, the associated vant Hoff entropy of unfolding, Δ*S*_vh_, and aggregation kinetics. Molecular dynamics simulations that included both glycine and the citrate buffer reveal complex competing interaction types involving all of the formulation components that explain the experimental observations. The glycine concentration dependence showed three distinct phases of mechanistic action in which glycine starts with preferential interactions that displace water from the hydration shell and then gradually displaces the increasingly tightly bound citrate molecules. Displacement of citrate leads to increased protein flexibility, as monovalent glycine interrupts the stabilizing effects of the multivalent citrate. As this displacement of citrate is unfavorable, the glycine added at higher concentrations gradually becomes preferentially excluded. Finally, glycine interacts with other protein surface sites but with no further displacement of water or citrate and leads to a reduction in protein flexibility. The impact of glycine and the mechanisms observed by MD simulation is consistent with the aggregation mechanism that is rate limited by partial unfolding to reveal APRs.

## Materials and Methods

### Fab Production

A33 Fab (C226S) was expressed from *Escherichia coli* strain W3110 in a Biostat Cplus 30 L fermenter (Sartorius Stedim, U.K.) and purified by protein G chromatography, gel filtration, and buffer exchange into formulations as previously described.^29^ Protein concentrations were determined by absorbance at 280 nm and an extinction coefficient of 1.4 cm^–1^·mL·mg^–1^ (66,329 mM^–1^ cm^–1^).

### Aggregation Kinetics

Aggregation kinetics were determined for 1 mg·mL A33 Fab in 10 mM sodium phosphate, pH 7, or in 20 mM sodium citrate, pH 4.5, with NaCl (0.028 mM at pH 7, or 0.01 mM at pH 4.5) to a final ionic strength of 50 mM, at 65 °C, for a range of formulations. At pH 7, formulations contained 40 mg/mL mannitol, 40 mg/mL sorbitol, 4% (w/v) Tween 80, 20 mg/mL glycine, or no excipient. At pH 4.5, formulations contained trehalose, sucrose, mannitol, sorbitol, Tween 20, Tween 80, arginine, or glycine at various concentrations.

Kinetics were determined as rates of monomer loss with monomer fraction determined by size exclusion chromatography-high-performance liquid chromatography (SEC-HPLC) as previously, at time points ranging from 0 to 8 days (pH 4.5) and from 0 to 60 days (pH 7). Monomer content was expressed as % monomer retained relative to an undegraded Fab standard. All measurements from degradation kinetics experienced a “dead time” of approximately 2 min between sampling and quenching by cooling prior to SEC analysis. This dead time was insignificantly relative to the first time point of 2 h and 8–60 day timecourses and so was ignored in the fitting. All curves were fit to the first order exponential decay in eq 1, where *M*_0_ is the initial monomer concentration, *k*_obs_ is the rate constant, *M* is the monomer retention, and *t* is the incubation time. Initial aggregation rate (*v*) was determined as *M*_0_*× k*_obs_ at *t* = 0.
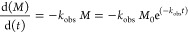
1

### Thermal Stability Analysis

Thermal stability analysis for the A33 Fab (C226S) formulations was determined from the change in intrinsic fluorescence as a function of temperature using the UNit instrument (Unchained Laboratories, U.K.). Each formulation was made in triplicate at 3 mg/mL A33 Fab in 20 mM sodium citrate, pH 4.5, with NaCl to a final ionic strength of 50 mM, and final glycine concentrations of 0–60 mg/mL. Samples were unfolded with a temperature ramp from 20–90 °C at 1 °C/min. The thermal transition midpoint temperature (*T*_m_) was determined from the barycentric mean (BCM) of protein intrinsic fluorescence spectra (280–460 nm), at each temperature, by fitting to eq 2 as previously,^29^ and then at the *T*_m_, Δ*S*_vh_ = Δ*H*_vh_/*T*_m_.
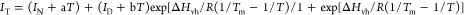
2where, *I*_T_, *I*_N_, *I*_D_ are the signal at temperature *T*, in the native state N, and in the denatured state D.

### All-Atom Molecular Dynamics Simulations

MD simulations were performed using Gromacs 5.0.4.^49^ and the CHARMM36m force field, using an A33 Fab homology model generated previously^47^ from template structure PDB: 1T3F. The partial charges and nonbonded parameter for zwitterionic glycine were modeled with CHARMM36m parameters in Gromacs. The citrate parameters were those already used in the CHARMM36m FF^50^ taking the 3^–^ charged species prevalent at pH 4.5. MD simulations used the TIP3P water model, LINCS algorithm for hydrogen bond constraints, and the Verlet cutoff scheme for van der Waals interactions with a cutoff distance of 1.2 nm. Particle-mesh Ewald (PME) method with a 1.2 nm cutoff distance was used for long-range electrostatics, and periodic boundary conditions were used.

MD simulations were performed in a cubic box of side 12.4 nm, at pH 4.5, with protonation states assigned to amino acid residues using PROPKA^51^ on the PDB 2PQR server.^52^ The maximum native length of the Fab is 7.4 nm, and so a box size of 12.4 nm ensured no protein–protein interactions across the boundaries. MD simulations were made charge-neutral with the addition of sodium and chloride counterions and then also increased to achieve 50 mM ionic strength. Citrate and glycine molecules were randomly inserted into the simulation box, with water extending at least 1.5 nm from the protein surface. The simulation composition is detailed in Table S1 (Supporting Information). Energy minimization (Steepest Descent and Conjugate Gradient) and 5 ns of equilibration (NVT and NPT) were performed prior to production MD simulations at 338 K and 1 atm. Production MD simulations were carried out for 60 ns, with configurations saved every 10 ps. Simulation jobs were sent to the UCL Grace high performance computing facility. Four independent production MD simulations for each formulation condition were performed, resulting in a total simulation time of 240 ns per formulation. Analyses were performed over the last 40 ns of the MD simulations (except for RMSD). Standard Gromacs tools were used unless stated. All analyses except principal component analysis (PCA) were calculated for each replica independently, and then the data were averaged and used to calculate standard errors. Statistical errors are represented by the standard error of the mean (SEM).

### Bulk Solution Analysis

Hydrogen bonds between glycine molecules were used to characterize the extent of glycine clustering using a 0.22 nm donor–acceptor (D–A) distance and 30° donor-hydrogen-acceptor (D–H–A) angle cutoff. A 0.35 nm d–a distance and 30° d–h–a angle cutoff was used to characterize citrate-glycine interactions.

### A33 Fab Surface Analysis

Preferential interaction coefficients were calculated from the MD simulations using radial distribution functions (RDFs) centered on the Fab center of mass, according to eq 3, as described previously^53^
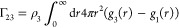
3where, equation ρ_3_ is the number density of the glycine, *g*_3_(*r*) and *g*_1_(*r*) is the RDF for glycine and water from A33 Fab. A 0.6 nm cutoff was used to define the local domain, which corresponds to approximately two hydration shells.

Buffer-Fab, glycine-Fab, and water-Fab hydrogen bonds were defined by a 0.35 nm *d*–*a* distance and 30° *d*–*h*–*a* angle cutoff. Contact frequencies of citrate and glycine were determined based on the minimum distance from A33 Fab using a 0.6 nm cutoff. The distribution of glycine N and O atoms around the heavy atoms of Fab charged, polar side chains and the backbone was shown by using RDFs. A 0.35 and a 0.6 nm cutoff were used to define the first and second water hydration shells, respectively. The average number of waters were calculated in each hydration shell.

### A33 Fab Structural Analysis

The root-mean-square deviation (RMSD) of A33 Fab backbone atoms relative to a reference structure was used to determine the convergence of the MD simulations. Formulations in the presence of 20 mM citrate only are referred to as GLY0, then with increasing glycine from 10 to 60 mg/mL, referred to as GLY10 to GLY60. The change in root-mean-square fluctuation (RMSF) for GLY10 to GLY60 relative to the GLY0 formulation was determined from the overall, backbone, and side chain RMSF values. Solvent accessible surface area (SASA) calculations were calculated using a probe radius of 0.14 nm. Intramolecular hydrogen bonds between backbone N–H–O atoms were calculated using a 0.35 nm D–A distance and 30° D–H–A angle cutoff. Secondary structure analysis of A33 Fab was determined using DSSP^54^ in Gromacs. Principal component analysis on A33 Fab C_α_ atoms was performed using Bio3D^55^ to extract the major motions of A33 Fab in the different glycine formulations. Simulation replicas were combined into a single concatenated trajectory prior to PCA. Changes in RMSF at the residue or global average level were calculated relative to those for Fab in 0 mg/mL glycine using eq 4.
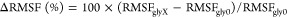
4

## Results and Discussion

### Glycine had the Largest Impact on A33 Fab Aggregation Kinetics

In previous work, we tested the stability of A33 Fab in nine different formulations, each with either no excipient, or one of eight single excipients, added into 10 mM sodium phosphate buffer, pH 7.^37^ Of these, 2% (w/v) glycine had the greatest increase in the thermal denaturation transition midpoint (*T*_m_) (see Table 1). We have now measured the aggregation kinetics (rate of monomer loss) at 65 °C for five of these formulations, including glycine, and observed that the increase in *T*_m_ correlated well to a decrease in aggregation kinetics at 65 °C (Supporting Information, Figure S1), consistent with our previous work that varied pH and ionic strength of formulations.^12^

**Table 1:** Experimental Stability Performance for a Range of A33 Fab Formulations^a^

formulation	[excipient](mg/mL)	*T*_m_ (°C)^b^ (±0.1) pH 7	ln *v* (*v* in % day^–1^) pH 7, 65 °C	*T*_m_ (°C) pH 4.5	ln *v* (*v* in % day^–1^) pH 4.5, 65 °C
no excipient	0	79.4	–0.51 (+0.3)	77.1 (1.0)	3.9 (+0.1)
trehalose	50	80.4			
	100				3.0 (+0.3)
	200				2.5 (+0.1)
sucrose	50	80.2			
	100				3.4 (+0.2)
	200				2.6 (+0.2)
mannitol	40	80.1	–0.99 (+0.5)		4.2 (+0.1)
	100				4.0 (+0.1)
sorbitol	40	80.3	–1.17 (+0.3)		4.2 (+0.1)
	100				3.6 (+0.1)
Tween 20	4	80.5			3.4 (+0.2)
	8				3.5 (+0.2)
Tween 80	4	80.1	–0.86 (+0.05)		
arginine	20	67.5			3.8 (+0.1)
	50				5.8 (+0.1)
glycine	10			79.1 (0.1)	
	20	81.6	–2.14 (+0.5)	79.6 (0.4)	4.1 (+0.1)
	30			82.2 (1.0)	
	40			82.2 (0.8)	
	50			83.2 (0.2)	2.2 (+0.2)
	60			83.6 (0.6)	

a ln (*v*) was calculated from the initial aggregation rates (*v*) determined as *M*_0_*× k*_obs_ at *t* = 0.

b Data from Barata et al.^37^ Studies at pH 7 were carried out in 10 mM sodium phosphate buffer. Studies at pH 4.5 were carried out in 20 mM sodium citrate buffer. Standard errors of the mean (SEM) are shown in parentheses. For ln *v*, only the positive error is shown.

Glycine was found to have the biggest stabilizing impact on both the *T*_m_ and the rate of monomer loss in A33 Fab compared to the other excipients. The study was extended to investigate the effects of broader excipient concentrations on aggregation kinetics. Our previous analysis of pH-dependent aggregation kinetics of A33 Fab showed that the protein aggregated more rapidly at pH 4.5, and on a time scale that was more convenient for study,^12^ and so aggregation kinetics (rate of monomer loss) for the new formulations were evaluated in 20 mM citrate buffer, pH 4.5 at 65 °C (Table 1). Again glycine was observed to have the greatest impact and also a strong concentration dependence, bringing the aggregation kinetics to the slowest of all conditions at 50 mg/mL (ln *v* = 2.2), and approximately 5× slower than in the absence of excipient (ln *v* = 3.9). However, the dependence of aggregation kinetics on glycine concentration was nonlinear given that the low concentration of 20 mg/mL glycine actually increased the kinetics slightly compared to at 0 mg/mL.

### Effect of Glycine Concentration on the Thermal Stability of A33 Fab

**Figure 1:**
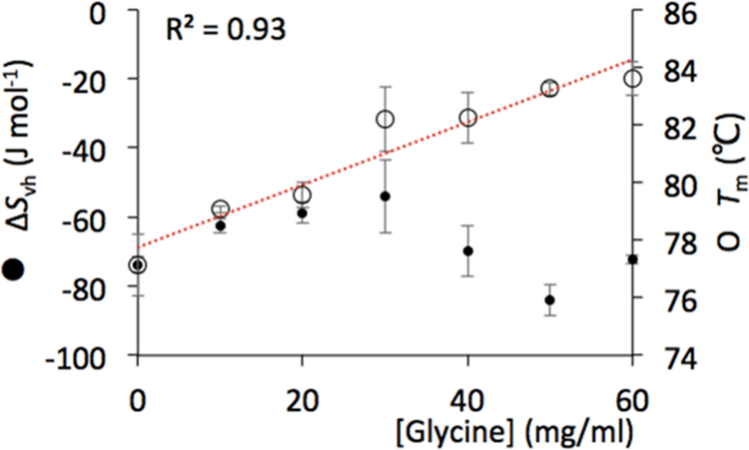
Effect of glycine concentration on the conformational stability of A33 Fab. The change in van’t Hoff entropies (Δ*S*_vh_) and the thermal transition midpoint temperatures (*T*_m_) for 3 mg/mL A33 Fab as a function of glycine concentration in 20 mM sodium citrate, pH 4.5, were determined from thermal unfolding profiles.

To further evaluate the stabilizing effects of glycine on A33 Fab, the *T*_m_ was measured using intrinsic protein fluorescence, in the presence of 20 mM citrate buffer, pH 4.5. Overall, the *T*_m_-values for A33 Fab ranged from 77.1 °C in the absence of glycine, to a maximum of 83.5 °C at 60 mg/mL glycine, as shown in Figure 1. The increase in *T*_m_ was essentially monotonic with the glycine concentration, indicating a gradual improvement in thermal stability upon the addition of glycine. Taken alone, these data would only require a single mechanism of action to explain them, such as preferential exclusion.

### Effect of Glycine Concentration on A33 Fab Unfolding Δ*S*_vh_

The entropy changes (Δ*S*_vh_) between native and denatured states were also determined for each formulation at the *T*_m_ using van’t Hoff analysis of the thermal denaturation curves. These revealed a more complex mechanism of action in terms of the impact of glycine on the A33 Fab structure. At lower bulk concentrations of glycine (between 0 and 30 mg/mL glycine), the Δ*S*_vh_ decreased in magnitude, which indicated the presence of more conformational states or greater flexibility overall under native conditions. However, from 30 to 50 mg/mL glycine, the trend altered direction, and the magnitude of Δ*S*_vh_ increased again, suggesting a regain in compactness or more contacts formed in the native ensemble relative to the denatured state. The magnitude of Δ*S*_vh_ then decreased again at 60 mg/mL (Figure 1).

Clearly, the stabilization of Fab with glycine was complex, such that while the *T*_m_ increased linearly, the underlying structural dynamics underwent several phases due to at least two different mechanisms, which were observed via changes in the apparent cooperativity of the thermal denaturation transition. Such a change in mechanism also appeared to be the cause of the nonlinear dependence of the aggregation kinetics on glycine concentration as described above, and so also the loss of correlation between *T*_m_ and aggregation kinetics. Increasing the glycine concentration from 0 to 20 mg/mL had very little impact on the aggregation kinetics at pH 4.5, yet these were much reduced at 50 mg/mL glycine. This trend more closely reflected the nonlinear dependence of Δ*S*_vh_ on glycine concentration, than the linear dependence of *T*_m_.

This observation was consistent with our previous analysis of single mutants of A33 Fab that reduced the native state flexibility (increased Δ*S*_vh_) with no change in *T*_m_, and yet slowed the aggregation kinetics.^48^ In another previous study the increased concentration of A33 Fab itself was found to produce a two-state transition from an open form to a more compact form of the protein which led to a decrease in aggregation kinetics at the higher protein concentration.^29^ Those effects could not be replicated by simple crowding agents such as dextran 40 or Ficoll 70, and were instead consistent with specific self-interactions of the protein. Similarly, the current nonlinear dependence of aggregation kinetics on glycine concentration also suggests a more complex stabilizing mechanism than simple crowding, such as through specific interactions of the excipient with the protein.

To elucidate any potential mechanisms further, we carried out detailed all-atom molecular dynamics simulations of the glycine formulations with all of the buffer and excipient components present.

### Deconvoluting the Stabilizing Mechanism of Glycine in Citrate Buffer Using MD Simulations

All-atom molecular dynamics simulations of A33 Fab were performed for 60 ns, as four repeats, in the presence of 20 mM citrate only (hereby referred to as GLY0) and 20 mM citrate with increasing glycine from 10 to 60 mg/mL (hereby referred to as GLY10 to GLY60), all at pH 4.5.

### Propensity of Glycine–Glycine and Citrate-Glycine Interactions Increased with Bulk Glycine Concentration

**Figure 2:**
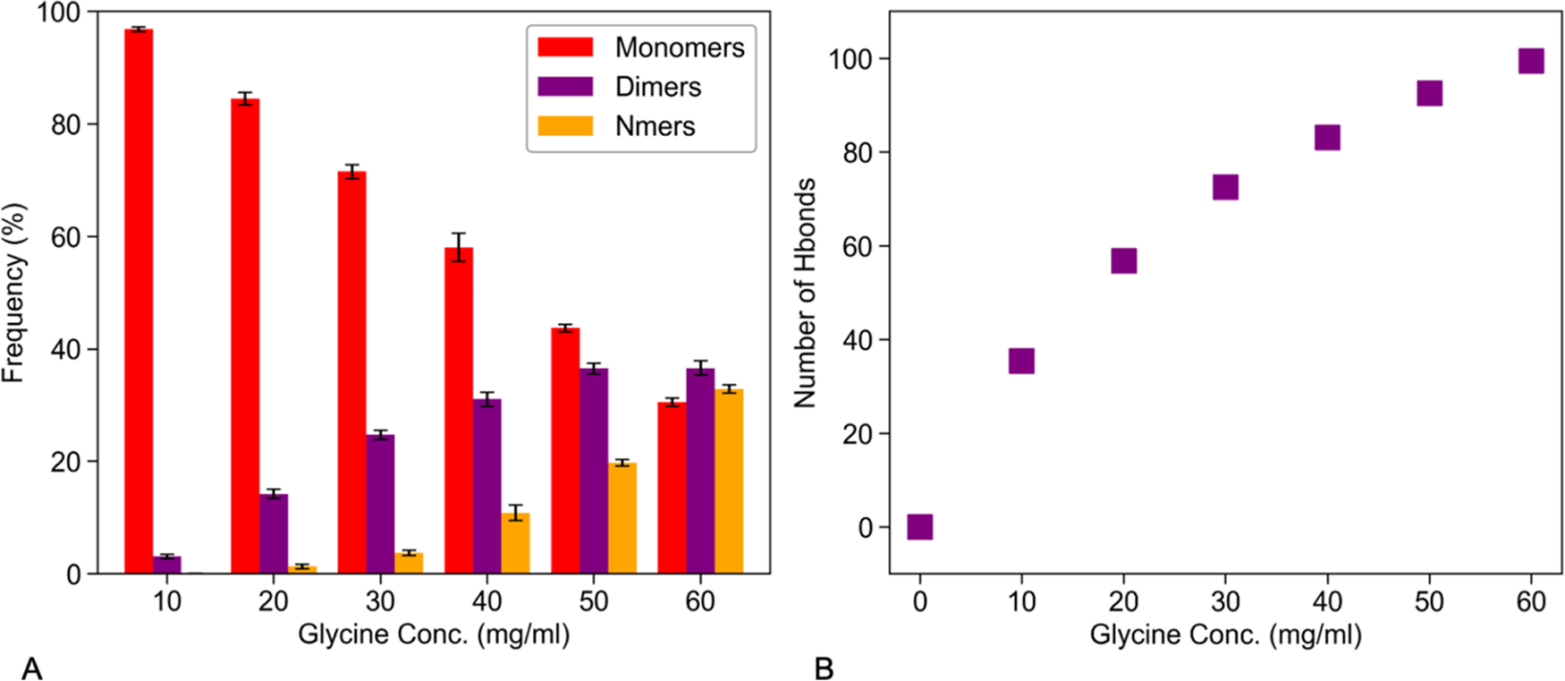
Concentration-dependent self-association of glycine, and interactions with citrate in MD simulations. (A) Monomeric fraction of glycine decreases, while dimer & Nmer populations increase with increasing bulk glycine concentration. (B) Number of hydrogen bonds formed between citrate and glycine as a function of the bulk glycine concentration.

To understand the excipient effects on A33 Fab in each formulation, we first characterized glycine–glycine and citrate-glycine interactions in our MD simulations by analyzing their hydrogen bonding, over the last 40 ns of simulation for each glycine concentration. The formation of hydrogen-bonded glycine clusters in aqueous solutions has been extensively explored using experimental and MD simulation methods.^56,57^ Here we defined glycine self-association based on the formation of a hydrogen bond between two glycine molecules. The fraction of glycine observed as monomers (no hydrogen bond), dimers (one hydrogen bond), and Nmers (more than one hydrogen bond) is shown in Figure 2A. The monomeric glycine population decreased from GLY10 to GLY60, while the fraction of dimers and Nmers increased. A snapshot of a typical glycine dimer, whereby the N–H forms a hydrogen bond with the oxygen on the adjacent glycine molecule, is shown in Figure S2A (Supporting Information).

Zwitterionic glycine can associate with other glycine molecules or with citrate via salt-bridge type interactions to form larger assemblies of glycine molecules. From our simulations, we found that interactions between citrate and glycine molecules increased with increasing bulk concentration of glycine Figure 2B. The most common interaction for the N–H on glycine to form one or two hydrogen bonds to bridge across two of the citrate carboxylate oxygens or hydroxyl moiety (Supporting Information, Figure S2B).

### Elucidating the Preferential Interaction/Exclusion Behavior of Glycine in the MD Simulations

**Figure 3:**
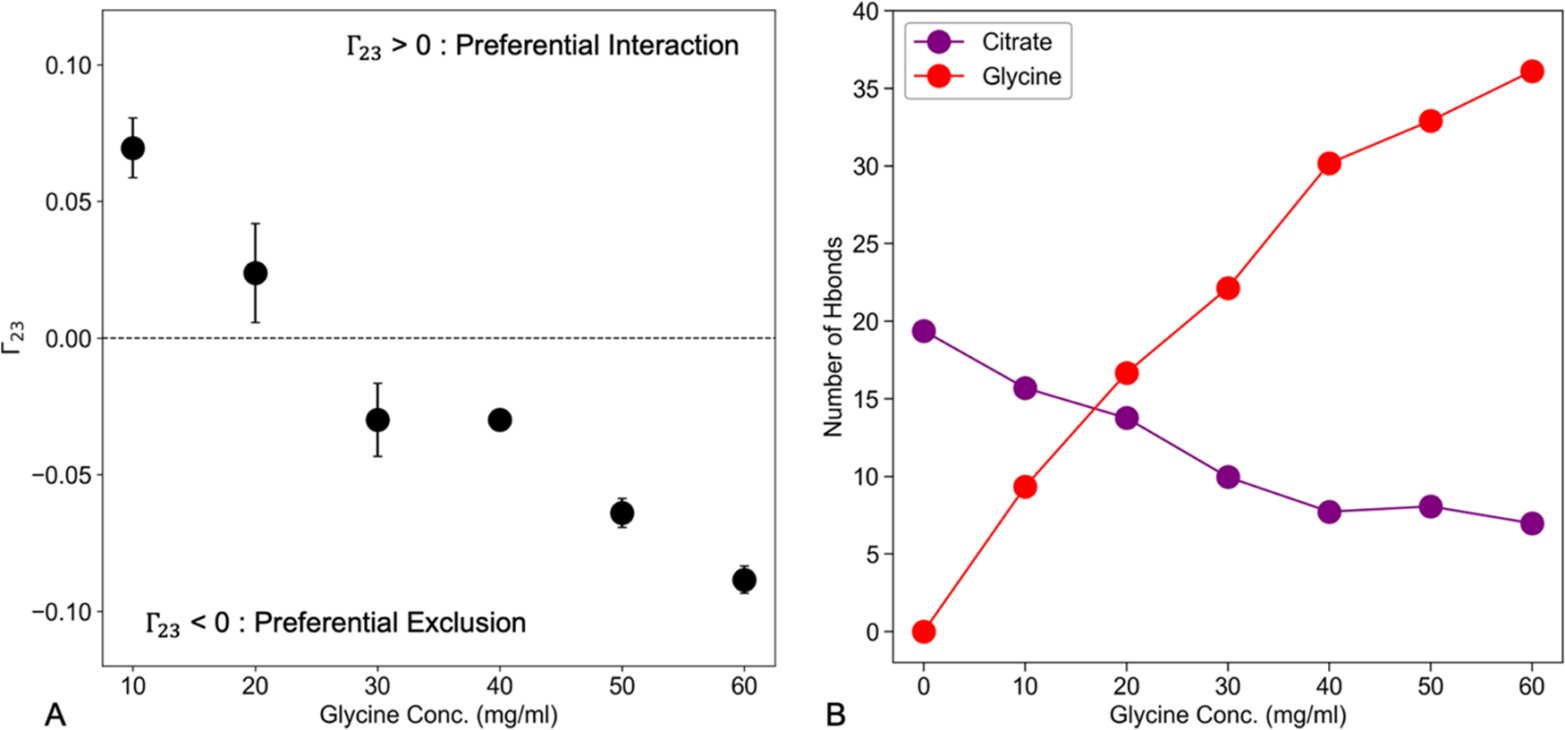
Quantifying the interactions of glycine and citrate with Fab during MD simulations. (A) Preferential Interaction Coefficient (Γ_23_) as a function of glycine bulk concentration. (B) Number of hydrogen bonds formed between excipients (citrate and glycine) and A33 Fab as a function of the bulk glycine concentration.

Preferential interaction infers that the protein prefers to interact with the excipient (i.e., a large excess of excipient in the local domain of the protein). Conversely, preferential exclusion refers to the preference of the protein to interact with water over the excipient (i.e., excipient is excluded from the protein surface). The preferential interaction coefficient (Γ_23_) at each glycine concentration (in the presence of citrate buffer) was calculated from the last 40 ns of MD simulations using eq 2. In the GLY10 and GLY20 simulations, glycine was found to be slightly preferentially bound (0 < Γ_23_ < 1) to the Fab. As the concentration of glycine was increased (GLY30 to GLY60), the Γ_23_-values became less than zero, and with increasing magnitude, indicating that glycine became preferentially excluded from the vicinity of A33 Fab (Figure 3A). The Γ_23_-value as a function of glycine bulk concentration shows a near linear trend, which is in agreement with other prominent osmolytes such as sucrose, trehalose, and sorbitol.^38^

The trend observed in Γ_23_-values can be explained in terms of the competition to attract glycine molecules by either the bulk solution or the protein surface. Self-interaction of glycine and the interaction between glycine and A33 Fab are the main driving forces that determine the number of glycine molecules accumulating near the A33 Fab surface. At low bulk glycine concentrations (GLY10 and GLY20), protein surface residues which interact favorably with glycine appear to become occupied as indicated by the positive Γ_23_-values. One would expect this as the number of glycine molecules in the bulk is small and the occupancy of the surface residues is relatively low. With an increase in bulk glycine concentration, there is an increase in glycine self-association but also potentially fewer sites freely available for protein interactions, leading to negative Γ_23_-values (preferential exclusion).

### Mapping the Interactions between Fab-Citrate and Fab-Glycine

Differences in the patterns of interaction of citrate and glycine with the A33 Fab surface residues were quantified by the number of hydrogen bonds formed (Figure 3B). Overall, the number of hydrogen bonds formed by glycine with Fab increased from GLY10 to GLY60, and generally correlated to the *T*_m_, but the slope began to decrease at between GLY40 to GLY60. This also reflects the shift toward preferential exclusion whereby the number of hydrogen bonds between glycine and the protein increases, but not as fast as the actual glycine concentration. Hence, the glycine concentration increased faster in the bulk solution than around the protein, leading to the decrease in Γ_23_.

**Figure 4:**
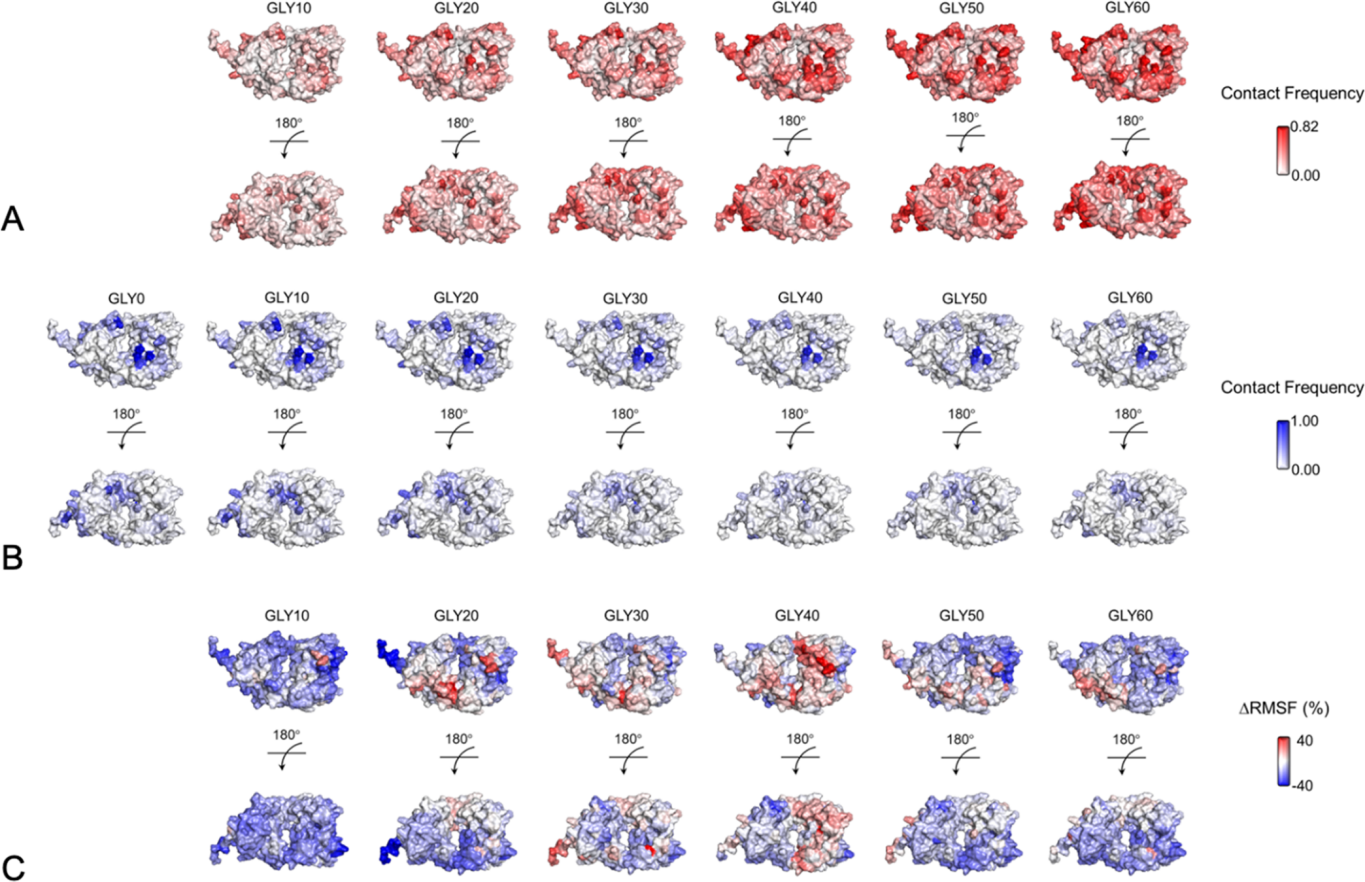
Surface mapping of Fab-Gly and Fab-citrate interactions, and the residue-level ΔRMSF. (A) Glycine contact frequencies mapped onto the Fab surface. (B) Citrate contact frequencies were mapped onto the Fab surface. (C) Residue level ΔRMSF (%) relative to 0 mg/mL Gly.

The trend in hydrogen bonds to the protein was also reflected in the glycine contact frequency values, as mapped onto the Fab surface (Figure 4A), which indicated that the available binding sites were becoming fully occupied at higher formulation conditions. The existence of both positively charged (−NH_3_^+^–) and negatively charged (−COO^–^–) functional groups allows glycine to bind to the side chains of positively and negatively charged and polar Fab residues. Backbone interactions were found to be less prominent.

In contrast, the number of citrate-Fab hydrogen bonds decreased from GLY10 to GLY60 (Figure 3B) as a result of glycine displacing the bound citrate molecules. This led to an increase in unbound citrate molecules in the solvent as well as the increase in those interacting with glycine in the bulk (Figure 2B). Interestingly, the glycine did not completely remove the citrate bound to Fab, which leveled off at GLY40 to GLY60, indicating that certain interactions between citrate and Fab had a higher affinity than the majority. Overall, the total number of hydrogen bonds to Fab increased (doubled at GLY60 compared to GLY0), indicating that there are many sites that become occupied by glycine, that were never occupied by citrate. This is potentially the result of the smaller size of glycine compared to citrate, but also the possibility of glycine to form interactions with neutral polar sites as well as both negatively and positively charged groups.

Associated with the binding of glycine to Fab, approximations of the number of water molecules in the first (<3.5 Å) and second (3.5–6 Å) hydration shells revealed that the initial glycine binding (GLY10) resulted in the displacement of 52 and 73% of water molecules in the first and second hydration shells, respectively, with little further change at higher glycine concentrations (Table 2). Most of the water in these hydration shells was not directly hydrogen bonded to the Fab surface, and the depletion of water reflected the preferential increase in the population of glycines within these hydration shells. There was also on average 22 hydrogen bonds to water molecules displaced (of the 819 formed at GLY0), and essentially no further change to GLY60 (Table 2). Given that the preferential interaction coefficient (Γ_23_) depends on the water and glycine density within the 6 Å cutoff defined by the second hydration shell, then these changes at 10 mg/mL glycine dominate the preferential interaction observed in Figure 3, and obscure the much smaller number of molecules involved in specific hydrogen bonding interactions to the protein surface.

**Table 2:** Number of Water Molecules in the Fab Hydration Shell and Hydrogen Bonds Formed from Glycine, Citrate, and Water to the Fab Surface^a^

glycine (mg/mL)	Fab-GLY	Fab-CIT	Fab-H_2_O	1^st^ shell H_2_O	2^nd^ shell H_2_O
0	0 (0)	19 (0.11)	819 (7.9)	3007	3170
10	9 (0.10)	16 (0.09)	797 (3.7)	1450	842
20	17 (0.14)	14 (0.10)	798 (6.7)	1447	830
30	22 (0.19)	10 (0.09)	800 (3.2)	1461	839
40	30 (0.15)	8 (0.06)	794 (4.1)	1450	820
50	33 (0.17)	8 (0.07)	801 (1.9)	1465	834
60	36 (0.18)	7 (0.07)	796 (2.8)	1439	813

a Standard errors of the mean are shown in parentheses.

### A33 Fab Reveals a Wide Range of Conformational States within the Native Ensemble

RMSD values calculated for the backbone atoms of all A33 Fab residues were used to explore the overall stability in different formulation conditions (Supporting Information, Figure S3). The distribution of RMSD values from the last 40 ns of the MD simulation demonstrated that A33 Fab exhibited a wide range of conformational states, but only within a generally narrow range of RMSD (0.3 to 0.57 nm). An overlay of the Fab conformations sampled during the MD simulations at each formulation condition is shown in Figure S3C (Supporting Information). The wide range of different conformational states potentially indicates the presence of local unfolding, conformational switching, or even the stabilization of selected regions. However, the dynamics observed were on short time scales only, and did not lead to any global unfolding or major conformational changes as also suggested by the small differences in *R*_g_, SASA and secondary structure content for each formulation (Supporting Information, Table S2). Therefore, the dynamics observed are likely to only represent those present within the native ensemble which can include localized partial unfolding events.

To identify the main types of conformational dynamics sampled during the simulations, Principal Component Analysis (PCA) was performed on the C_α_ atoms of A33 Fab. The first three principal components (PC1 to PC3) described more than 60% of the total variance observed in Fab at each formulation condition (Supporting Information, Figure S4). The motions captured by PC1 corresponded to the well-documented hinge-bending motion about the flexible linker, which results in a change in the “elbow” angle. A typical range for this angle spans between 117 and 227°.^58^ PC2 represented the Fab variable domain (heavy and light chain) twisting about the flexible linker, and PC3 represented the flapping motion of the hinge region in the C_H_1 domain (Supporting Information, Figure S4). The value of each PC remained the same across the formulation series, except that the total variance explained decreased significantly in GLY20. This indicated that for GLY20 a wider range of alternative PCs were populated, perhaps consistent with the increased flexibility of the protein inferred experimentally from the peak value of Δ*S*_vh_ (Figure 1).

### Examining the Flexibility of A33 Fab in Each Formulation

The flexibility of a protein can influence its stability, while local unfolding events are generally thought to play a major role in aggregation due to exposure of critical regions of protein more susceptible to forming cross-β sheets, so-called aggregation-prone regions (APRs) as described above. Thus, it was useful to evaluate the impact of glycine on the flexibility of Fab, particularly at the surface residues.

**Figure 5:**
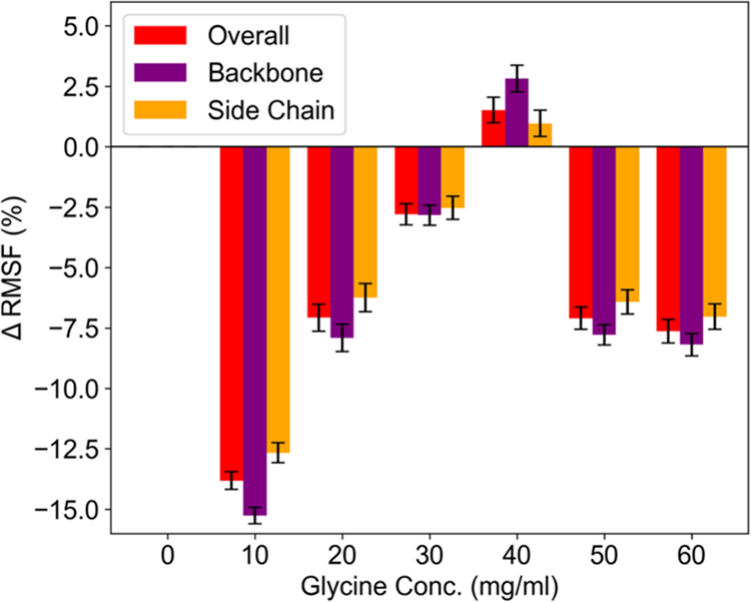
Global average ΔRMSF (%) as a function of glycine concentration for the whole protein, backbone, or side chain atoms. RMSF at each condition is the global average RMSF for all residues of Fab.

To identify Fab regions with altered flexibility due to the presence of glycine, we analyzed the residue-level change in RMSF relative to the GLY0 case in citrate buffer alone, whereby a positive ΔRMSF denotes an increase in flexibility. The dependence of RMSF on glycine concentration was nonlinear and reflected the mechanistic changes in the interactions between glycine and the protein surface as the concentration increased (Figure 5). The changes in RMSF also resembled the glycine concentration dependence of Δ*S*_vh_ (Figure 1) although the glycine concentrations with maximal flexibility (RMSF) and broader native ensemble (Δ*S*_vh_) differed slightly at 40 and 30 mg/mL respectively. Therefore, the events observed in the simulations may be sufficient to explain the changes in the Δ*S*_vh_.

The global average RMSF decreased by 15% upon initial addition of glycine (GLY10) (Figure 5), then increased from GLY10 to GLY40, reaching essentially the same level as in the absence of glycine, before decreasing again at GLY50 and GLY60 (Figure 5). The initial large change in RMSF at 10 mg/mL glycine occurred with nine glycine molecules forming hydrogen bonds to the Fab surface, the displacement of 52% of ∼3000 water molecules from the first hydration shell, and loss of 3 (of 19) citrate molecules hydrogen-bonded to the Fab surface. The binding of glycines, and the displacement of water from the hydration shell and protein surface led to the rigidification of the protein for the short-time scale (ps-ns) motions as accessed by MD simulations. This must have offset the impact of displacing three citrate molecules which would otherwise have been destabilizing as described below.

The displacement of citrate at 10–40 mg/mL glycine gradually increased the flexibility of the protein. Indeed there was a linear correlation between RMSF and the total number of glycine hydrogen bonds to Fab with an *R*^2^ = 0.98 in the concentration range 10–40 mg/mL glycine. An equally good but inverse correlation was observed for the number of citrate hydrogen bonds to the Fab. The citrate molecules displaced as glycine was increased from 10–40 mg/mL (133–533 mM) were presumably bound with higher affinity than glycine given that citrate was present in the buffer at only 20 mM. These results suggested that glycine (at 10–40 mg/mL) replaced citrate through mass action, but that the higher-affinity citrate molecules had been able to stabilize motions at the surface of Fab more than by the glycine that replaced them.

In principle, both the higher affinity and rigidifying influence of citrate would derive from its potential to simultaneously form more hydrogen bonds (7 hydrogen bondable atoms) to the protein surface than can glycine (3 hydrogen bondable atoms).

As the displacement of citrate generally led to increased flexibility, it could not explain the rigidification at 50 mg/mL glycine and above. Furthermore, no additional citrate was displaced from Fab at these concentrations of glycine. Instead, the decrease in RMSF at the highest glycine concentrations occurred as glycine bound to the Fab surface without displacing water or citrate. This indicates that these interactions were generally weaker and yet exerted sufficient collisional or steric impact on the protein surface to decrease its flexibility. This embodies a stabilizing macromolecular crowding mechanism within the regime of preferential exclusion, such that while the glycine excipient prefers to self-interact and form oligomers or to interact with citrate in the buffer, it still cannot avoid interactions with the protein surface which limit its conformational freedom.

**Figure 6:**
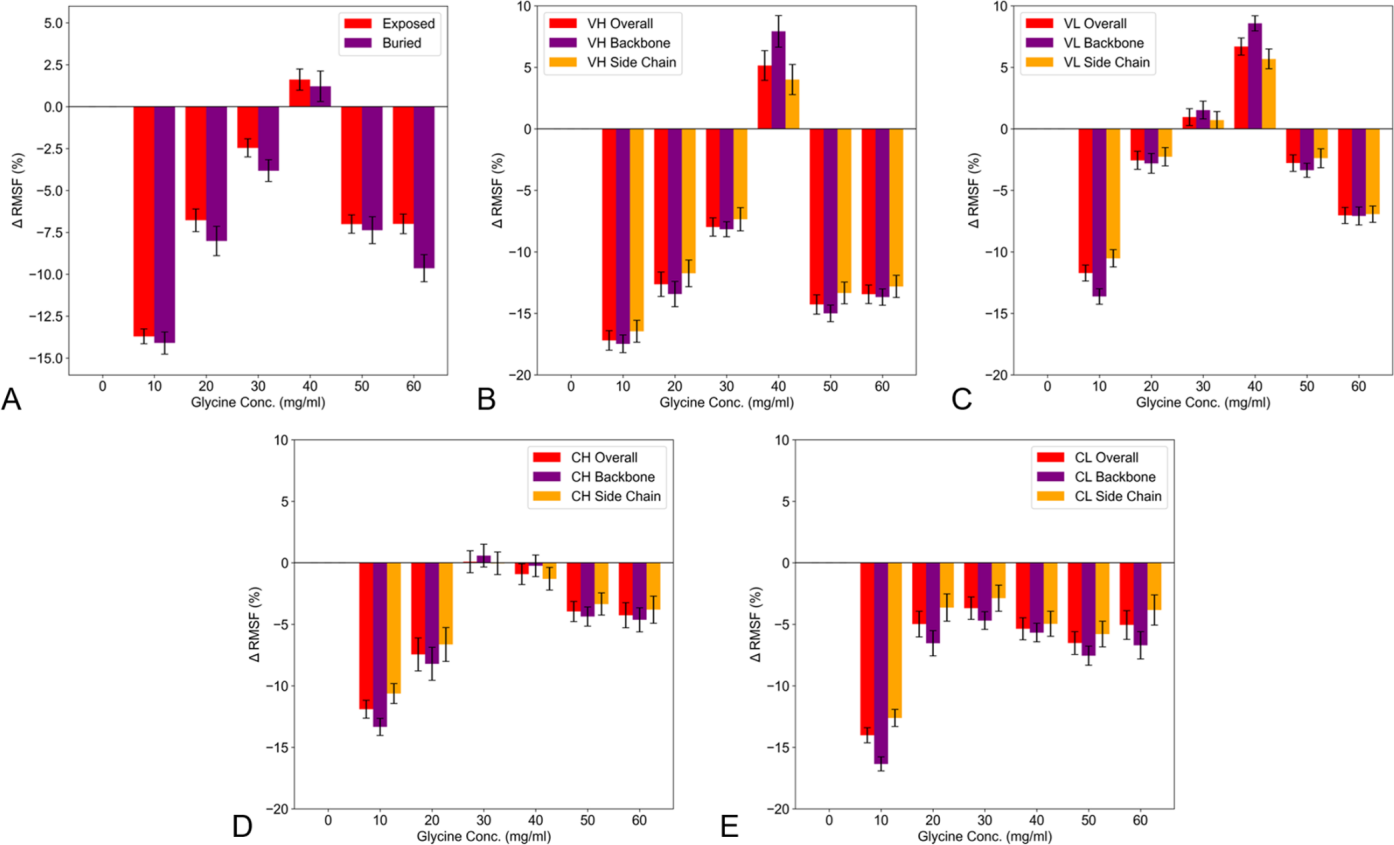
Global average ΔRMSF (%) as a function of glycine concentration by location within the protein. (A) Exposed and Buried A33 Fab residues. (B–E) Overall, backbone, and side chain for V_H_ (B), V_L_ (C), C_H_1 (D), C_L_ (E) domain residues.

The distribution of ΔRMSF values revealed that approximately 89% of Fab residues demonstrated lower flexibility at GLY10, thus, binding of the excipient impacted the flexibility of the whole protein. This number decreased dramatically to only 30% at GLY40 (Supporting Information, Figure S5). The ΔRMSF values were mapped to the Fab structure in Figure 4C which shows how the changes in ΔRMSF were localized to the various structural domains. Overall, buried residues were less flexible than surface residues but still changed in flexibility in a similar trend to the surface residues (Figure 6A). The variable domains varied in ΔRMSF with a similar trend to the overall protein (Figure 6 B,C), with a peak in flexibility at 40 mg/mL glycine that was greater than that at 0 mg/mL glycine. However, key differences emerged in the residues from the C_H_1 and C_L_ domains. For C_H_1 the flexibility peaked at 30 mg/mL glycine and only matched the flexibility at 0 mg/mL glycine (Figure 6D). For the C_L_ domain, the flexibility also peaked at 30 mg/mL glycine, but was largely the same from 20–60 mg/mL glycine (Figure 6E).

### Location of Citrate Molecules and Their Displacement from the A33 Fab Surface

**Figure 7:**
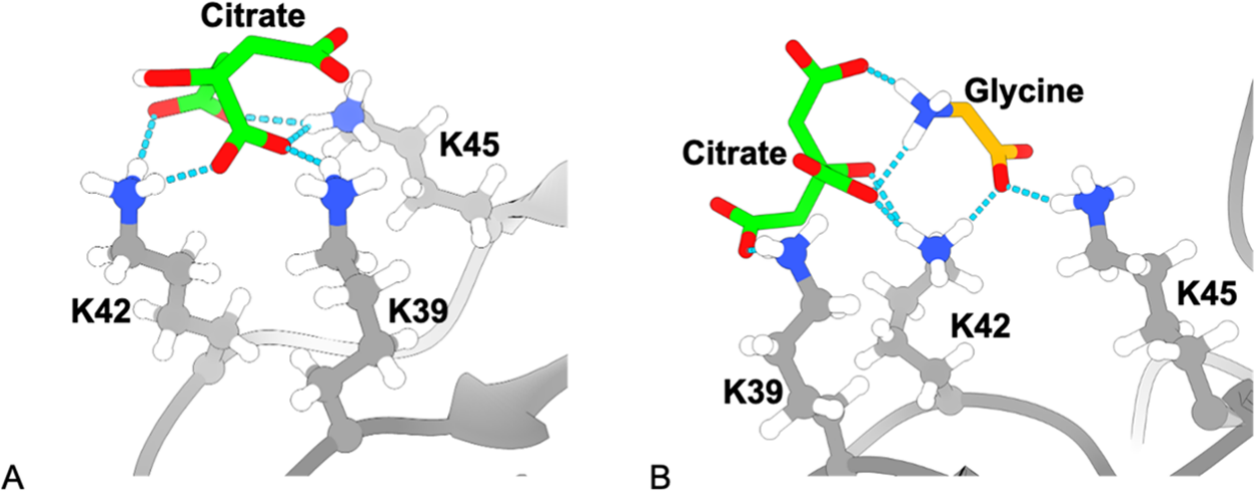
Snapshots of citrate and glycine interacting with the K39, K42, and K45 hotspot. (A) 0 mg/mL glycine. (B) 20 mg/mL glycine. At 20 mg/mL, interactions with both citrate and glycine are observed simultaneously.

The citrate molecules were randomly inserted into the simulation boxes for each replica and formulation. After the simulations, citrate was found to interact predominantly with lysine residues. The citrate molecules bound to Fab at 0 mg/mL glycine were then differentially displaced as the glycine concentration was increased. At the highest glycine concentrations, seven hydrogen bonds to citrate remained difficult to displace (Table 2), and were derived mainly from residues K39, K42, and K45, with some contributions from K419 and E81 (Supporting Information, Figure S6). These first three residues, along with E81, formed a single structural hotspot on the protein surface and interacted via seven hydrogen bonds to a single citrate molecule (Figure 7A). This hotspot was observed in all simulations, and maintained an occupancy of at least 60% for each hydrogen bond, even at 60 mg/mL glycine. This translated into occasional (40%) breaking of each hydrogen bond, but essentially 100% occupancy of the overall hotspot site in all conditions. During the breaking of these hydrogen bonds, which increased with glycine concentration, glycine interacted with them and caused their RMSF to increase. This RMSF increase was linked to the transition from a single citrate molecule bound and bridged across all residues, to a complex of citrate and glycine cooccupying the site (Figure 7B). The latter would be entropically weaker.

This site was one of three previously identified by molecular docking in A33 Fab, to be a binding hotspot for excipients.^37^ This particular site docked well with sucrose, trehalose, mannitol, sorbitol, polysorbate 20, and polysorbate 80, but not with the amino acids glycine or arginine. This may be due to repulsion between the positive charges on the lysine side chains and on the two amino acids in the Zwitterionic form, as well as on the arginine side chain. By contrast, citrate carries only negative charges and so would be expected to bind tightly, as observed in the MD simulations.

The differences in ΔRMSF profiles between the Fab domains (Figure 6) reflected the changes in citrate interactions for each domain, consistent with the role of citrate displacement by glycine leading to increased ΔRMSF. The V_L_ and V_H_ domains which had similar ΔRMSF profiles, shared an interaction to one citrate via K257(V_L_) and K103(V_H_) which was fully displaced at 20 mg/mL glycine. The V_H_ domain additionally had a strong interaction with citrate in a single cluster (Figure 7A) that was only partially displaced and remained >50% occupied even at 60 mg/mL glycine. The V_L_ domain had one other residue (E215) in close proximity (8 Å) to the strong citrate binding cluster in V_H_, which persisted at 30 mg/mL to 50% of the occupancy observed at 10 mg/mL and was only lost at 40 mg/mL.

The C_H_1 domain had a similar overall ΔRMSF profile to the variable domains, except that the ΔRMSF remained <0 at 40 mg/mL glycine. Two residues bound citrate to >50% of their original occupancy until the binding was lost in both cases at 30 mg/mL glycine. Thus, there was no displacement of citrate at 40 mg/mL glycine, resulting in the observation of no further increase in ΔRMSF. By contrast, the C_L_ domain showed no change in ΔRMSF at above 20 mg/mL glycine. That domain lost citrate interactions through four residues, mostly by 10 mg/mL glycine. One further residue (K419) in the C_L_ domain persisted with citrate binding up to 40 mg/mL.

## Conclusions

While thermal transition midpoint (*T*_m_) is often used for rapid early stage formulation screening, it is correlation with aggregation kinetics during long-term storage becomes poor once a certain level of thermal stability has been achieved.^12,13^ Therefore, to create a more streamlined approach to formulation, it is important to gain improved understanding of the mechanisms by which formulation excipients stabilize proteins against aggregation. The aggregation mechanism and kinetics of A33 Fab are mostly sensitive to native ensemble dynamics as previously revealed from Δ*S*_vh_ values of protein variants,^48^ and also from a detailed evaluation of the complex concentration dependence of A33 Fab aggregation kinetics.^29^ The glycine concentration dependence of *T*_m_ was almost monotonic. However, those for Δ*S*_vh_ and aggregation kinetics were more complex and again revealed that these two features are the most closely linked. This suggested it would be useful to elucidate the mechanisms by which glycine influences the native protein ensemble dynamics as an important controlling factor for the aggregation kinetics.

Molecular dynamics simulations of A33 Fab at each concentration of glycine revealed multiple molecular-level events that could contribute to the observed behaviors of Δ*S*_vh_ and aggregation kinetics. First, it revealed potentially important interactions between the glycine molecules, and also between glycine and the citrate buffer components, both in bulk solution and bound to the protein surface. This suggests the importance of adding buffer components into MD simulations, consistent with their known (de)stabilizing role in real formulations.

Glycine underwent a transition from preferential interaction with the protein surface at low concentrations to preferential exclusion at higher concentrations. This transition was driven by a change in the type of interactions that glycine could form with the Fab surface and also the type of molecules (hydration shell water, bound water, or bound citrate) that it needed to displace. At low concentrations, the glycine displaced 52% of the water in the first hydration shell as well as a small number of bound water and citrate molecules. The hydration shell water displacement was the main contributor to the preferential interaction observed. The resulting impact on the protein was a sharp decrease in RMSF which was a measure of protein flexibility, at least on the short time scales explored by MD. The Δ*S*_vh_ measurements did not show a similar loss in flexibility, and actually had a small increase, while the aggregation kinetics were unaffected, and so the experimental native ensemble must have been increased through conformational changes and dynamics that operate on longer time scales than MD, and also forming structural states that are not more aggregation prone than the average state.

At higher concentrations, glycine binding to the protein surface became more thermodynamically unfavorable, requiring the displacement of tightly bound citrate molecules. At this stage, glycine became preferentially excluded while it also readily formed dimers and higher ordered oligomers in the bulk solvent. Displacement of citrate which has the seven potential hydrogen bondable atoms, with glycine which has just three, led to an increased flexibility as measured by RMSF. This increase in flexibility was also observed in the Δ*S*_vh_ measurements, although the latter may include longer time scale mechanisms in addition to the short time scale events simulated by MD.

Finally, at ≥50 mg/mL, glycine continued to interact with the protein surface, but in locations not already occupied by water or citrate. The inability to displace further water and few citrate molecules suggest that glycine interactions with the protein surface were becoming less favorable thermodynamically. The RMSF and Δ*S*_vh_ measurements both showed a decrease in flexibility, and coincided with the slowed aggregation kinetics. Our results indicate that preferential exclusion is driven potentially by interactions between excipients and buffer components in the bulk solvent, along with increasingly unfavorable interactions available with the protein surface. The strong impact on protein dynamics and aggregation kinetics at high glycine concentrations indicates that the stabilizing mechanism was largely a macromolecular crowding effect.

This work suggests that Δ*S*_vh_ and the aggregation kinetics may be partially driven by short-time scale dynamics, but that a fuller explanation of the molecular mechanisms may require longer time scales to be explored, for example using enhanced sampling methods. However, the MD has shed useful insights into the mechanisms underpinning preferential interactions and preferential exclusion regimes, and also into the short time scale dynamics of A33 Fab, in a complex glycine excipient and citrate buffer system. This has laid important foundations for extending toward longer time scale studies. Although we already know that under most conditions studied the A33 Fab aggregation is rate limited by partial unfolding dynamics in the monomer, it may also be useful in future work to explore the impact on protein–protein interactions with multiprotein simulations.

## Supporting Information Available

Correlation between thermal transition midpoints and aggregation kinetics; snapshots of the interactions between glycine molecules and citrate; effect of glycine on Fab A33 backbone RMSD; impact of glycine on overall A33 Fab structure parameters calculated from MD simulations; principal component analysis on concatenated MD trajectories from each formulation condition; distribution of the ΔRMSF values for Fab A33 residues; local citrate hydrogen bond occupancies at each formulation condition (PDF)
