# Supplementary material for: Molecular Dynamics Simulations Reveal How Competing Protein–Surface Interactions for Glycine, Citrate, and Water Modulate Stability in Antibody Fragment Formulations
Source: Mol Pharm. 2024 Oct 21;21(11):5497–509. doi: 10.1021/acs.molpharmaceut.4c00332 (PMC11539065; doi:10.1021/acs.molpharmaceut.4c00332)
Supplement: Supplementary file 1 — mp4c00332_si_001.pdf [file mp4c00332_si_001.pdf]

Supplementary information

Molecular dynamics simulations reveal how competing protein-surface interactions for glycine, citrate and water modulate stability in antibody fragment formulations

Akash Pandya[1], Cheng Zhang[1], Teresa S. Barata[2], Steve Brocchini[2], Mark J. Howard[3], Mire Zloh[2], Paul A. Dalby[1*]

[1]Department of Biochemical Engineering, University College London, Gower Street, London, WC1E 6BT, UK
[2]School of Pharmacy, University College London, 29-39 Brunswick Square, London, WC1N 1AX, UK
[3]School of Chemistry, University of Leeds, Leeds, LS2 9JT, UK

Table S1. Fab MD simulation compositions and box dimensions for all formulations.

Glycine Conc. (mg/ml)	Cubic Box Dimension	Number of glycine molecules	Number of citrate molecules	Number of water molecules	Total number of atoms
0	12.4 nm	0	23	60437	188503
10		154		59803	188141
20		308		59159	187749
30		462		58513	187351
40		616		57864	186944
50		770		57219	186549
60		924		56607	186253

Note: the longest distance across the Fab is 7.4 nm.

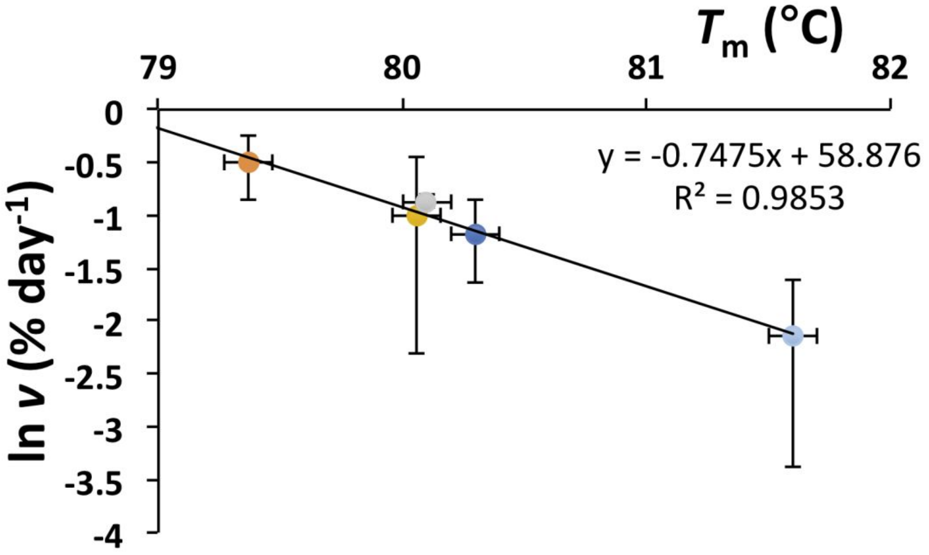

Figure S1. Correlation between thermal transition midpoints and aggregation kinetics at 65 °C for A33 Fab in five formulations in 10 mM sodium phosphate, pH 7. Formulations contained (yellow) 40 mg/ml mannitol, (dark blue) 40 mg/ml sorbitol, (grey) 4% (w/v) Tween 80, (light blue) 20 mg/ml glycine, or (orange) no excipient.

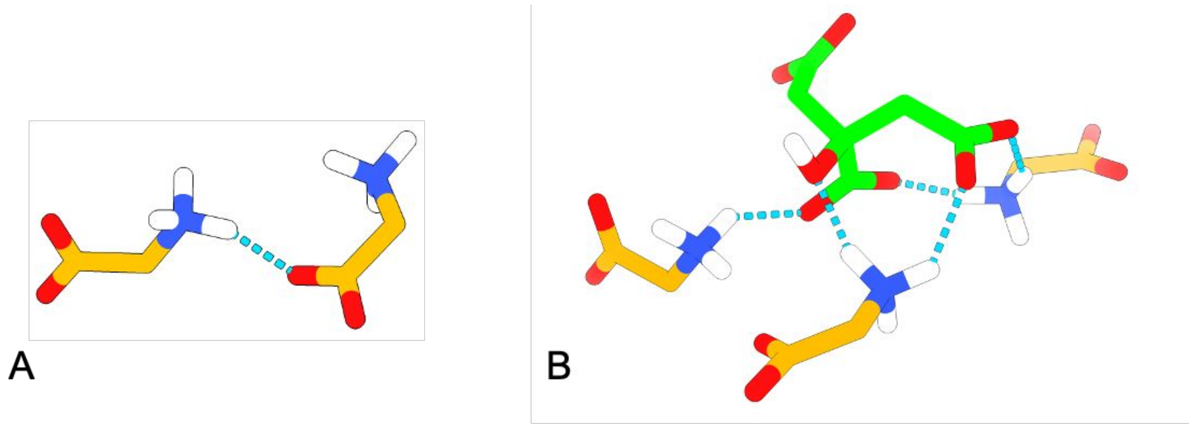

Figure S2. Snapshots of the interactions between A) two glycine molecules, and B) citrate and glycine molecules. A) Hydrogen bond shown between the amino hydrogen and the carboxylate oxygen of an adjacent glycine molecule. B) Hydrogen bond shown between the citrate carboxylate oxygen atoms and the glycine amino hydrogen atoms

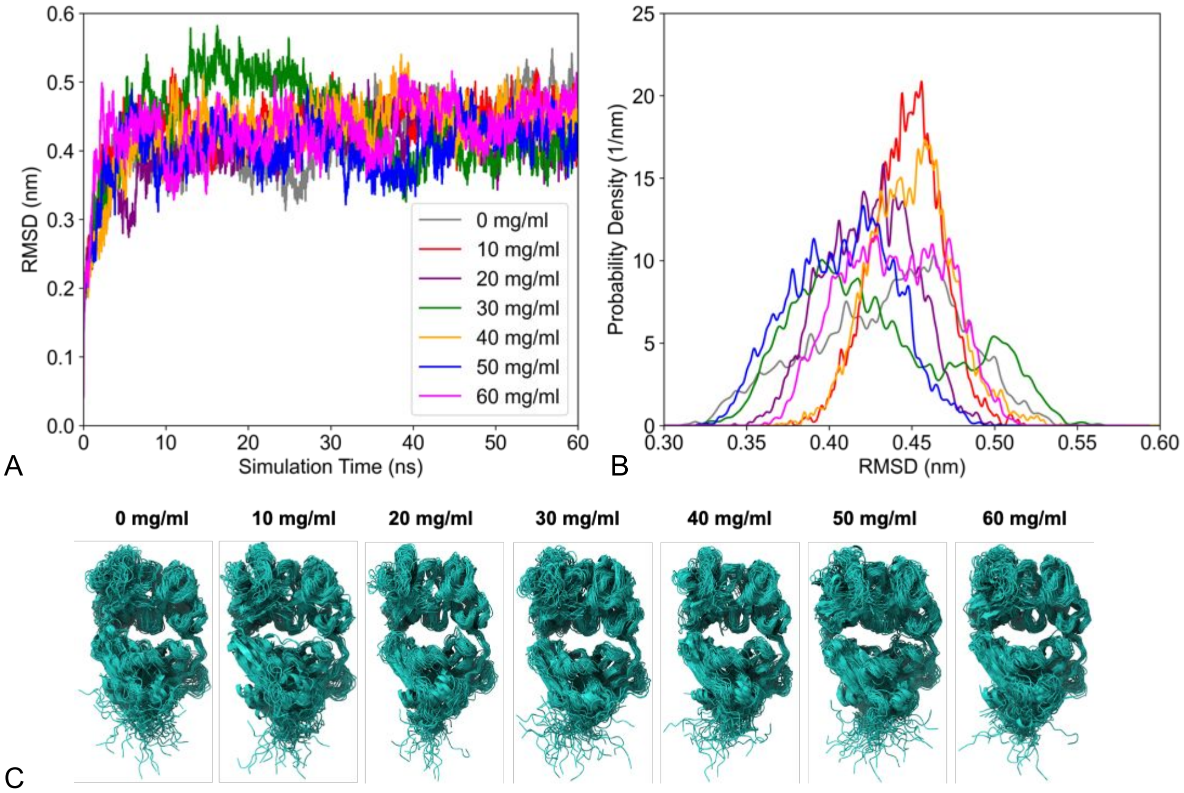

Figure S3. Effect of glycine on Fab A33 backbone RMSD. A) as a function of simulation time. B) Probability Density of backbone RMSD values from the last 40 ns of simulation for each formulation condition. C) Snapshots of Fab from simulations for each formulation condition from 0-60 mg/ml glycine in 20 mM citrate pH 4.5.

Table S2. Impact of glycine on overall A33 Fab structure parameters calculated from MD simulations.

**Formulation**	**R_g (nm)**	**Non-polar SASA (nm^2)**	**Total SASA (nm^2)**	**Alpha Helix (%)**	**Beta Sheet (%)**	**Turn (%)**
GLY0	2.49	40.2 ± 1.17	219 ± 0.12	2.5	46.0	12.0
GLY10	2.50	38.4 ± 0.38	216 ± 0.08	2.8	47.0	12.8
GLY20	2.51	39 ± 0.84	215 ± 0.12	2.8	45.8	12.3
GLY30	2.51	39 ± 0.84	218 ± 0.12	2.5	46.8	12.3
GLY40	2.51	38.9 ± 0.64	217 ± 0.09	2.5	47.3	12.8
GLY50	2.52	39.2 ± 0.34	220 ± 0.10	2.8	46.3	12.5
GLY60	2.50	38.4 ± 0.78	216 ± 0.09	2.5	46.5	12.3

R_g - Radius of gyration. Values calculated from last 40 ns of each simulation and averaged from four repeats. Errors shown are SEM.

	Alpha Helix Probability					
	Traj1	Traj2	Traj3	Traj4	Average	Stdev
GLY0	0.02	0.03	0.03	0.02	0.025	0.0058
GLY10	0.03	0.02	0.03	0.03	0.028	0.005
GLY20	0.03	0.02	0.03	0.03	0.028	0.005
GLY30	0.03	0.02	0.02	0.03	0.025	0.0058
GLY40	0.03	0.02	0.02	0.03	0.025	0.0058
GLY50	0.03	0.03	0.02	0.03	0.028	0.005
GLY60	0.02	0.03	0.02	0.03	0.025	0.0058

	Beta Sheet Probability					
	Traj1	Traj2	Traj3	Traj4	Average	Stdev
GLY0	0.46	0.46	0.45	0.47	0.46	0.0082
GLY10	0.47	0.48	0.46	0.47	0.47	0.0082
GLY20	0.45	0.47	0.45	0.46	0.46	0.0096
GLY30	0.46	0.46	0.47	0.48	0.47	0.0096
GLY40	0.46	0.48	0.47	0.48	0.47	0.0096
GLY50	0.45	0.46	0.47	0.47	0.46	0.0096
GLY60	0.46	0.48	0.47	0.45	0.47	0.013

	Turn Probability					
	Traj1	Traj2	Traj3	Traj4	Average	Stdev
GLY0	0.12	0.12	0.11	0.13	0.12	0.0082
GLY10	0.13	0.14	0.13	0.11	0.13	0.013
GLY20	0.13	0.12	0.12	0.12	0.12	0.005
GLY30	0.13	0.12	0.12	0.12	0.12	0.005
GLY40	0.13	0.12	0.13	0.13	0.13	0.005
GLY50	0.12	0.12	0.13	0.13	0.13	0.006

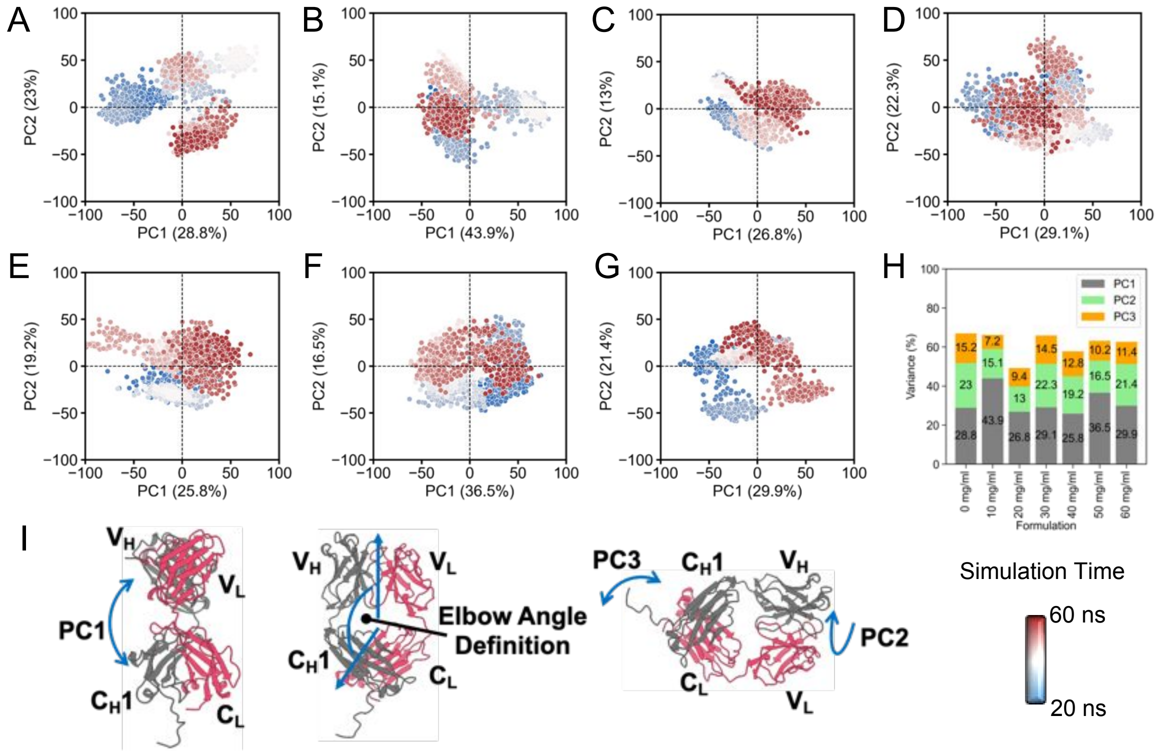

Figure S4. Principal Component Analysis on concatenated MD trajectories from each formulation condition. A-G) PC1-PC2 conformer plots for each formulation condition in order of increasing [glycine]. **H)** The variance (%) contribution made by the first three principal components at each condition (0-60 mg/ml glycine). **I)** A schematic depicting the major motions (represented by blue arrows) of Fab A33 corresponding to the first three principal components.

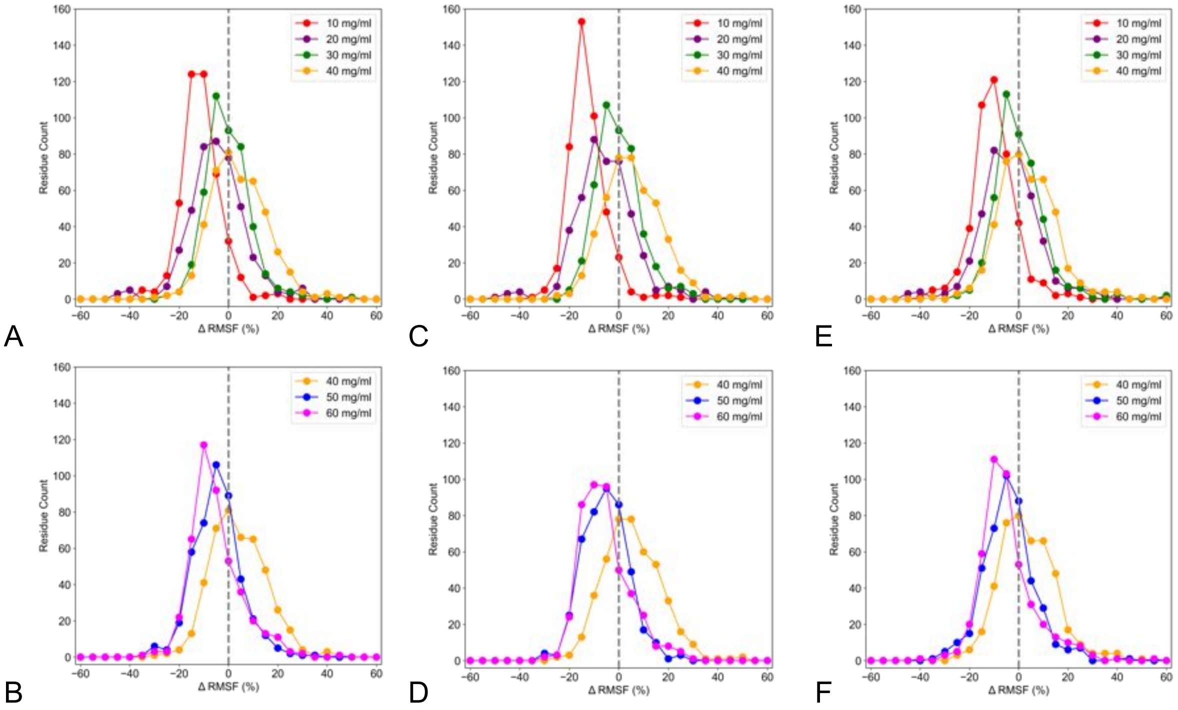

Figure S5. Distribution of the ΔRMSF values for Fab A33 residues.
A&B) Overall ΔRMSF for Fab in 10-40 mg/ml glycine **(A)** and 40-60 mg/ml glycine **(B).**
C&D) Backbone ΔRMSF for Fab in 10-40 mg/ml glycine **(C)** and 40-60 mg/ml glycine (**D**). **E&F)** Side chain ΔRMSF for Fab in 10-40 mg/ml glycine (**E**) and 40-60 mg/ml glycine (**F**).

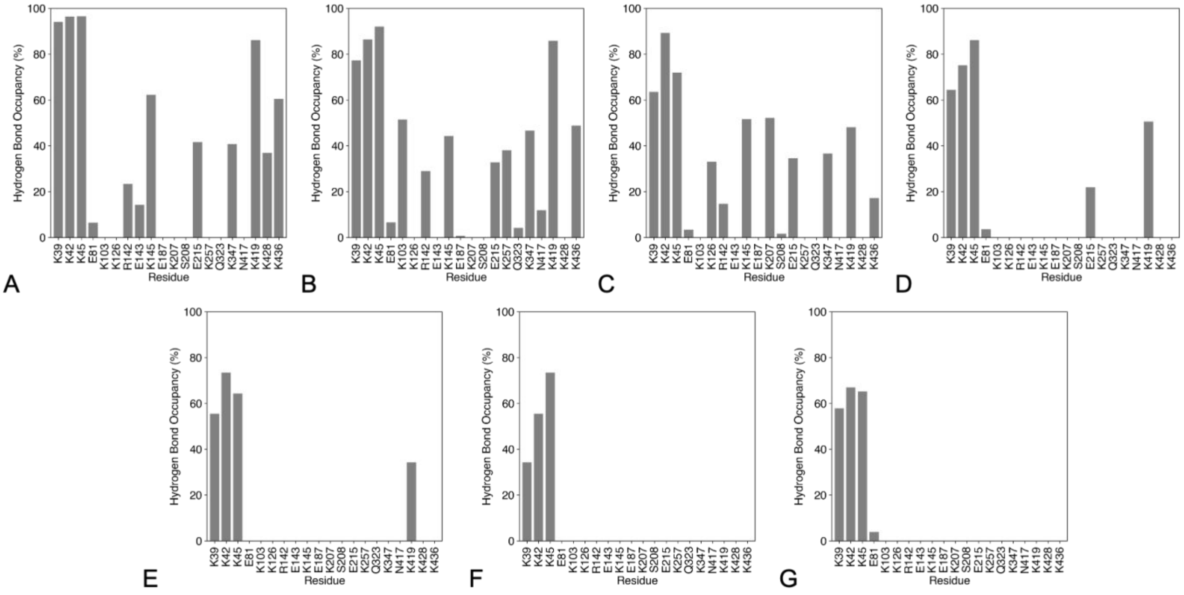

Figure S6. Local citrate hydrogen bond occupancies at each formulation condition. (A) 0 mg/ml glycine (B) 10 mg/ml glycine (C) 20 mg/ml glycine (D) 30 mg/ml glycine (E) 40 mg/ml glycine (F) 50 mg/ml glycine and (G) 60 mg/ml glycine.
